# A novel method for multiple bowel injuries: a pilot canine experiment

**DOI:** 10.1186/s13017-017-0155-0

**Published:** 2017-09-15

**Authors:** Jun Ke, Weihang Wu, Nan Lin, Weijin Yang, Zhicong Cai, Wei Wu, Dongsheng Chen, Yu Wang

**Affiliations:** 1 Department of Gastroenterology, Dongfang Hospital, Xiamen University, Fuzhou, Fujian 350025 China; 2 Department of General Surgery, Dongfang Hospital, Xiamen University, Fuzhou, Fujian 350025 China; 3 Department of Anesthesiology, Dongfang Hospital, Xiamen University, Fuzhou, Fujian 350025 China

**Keywords:** Intestinal injury, Ligation, Reconnecting, Endotoxin translocation

## Abstract

**Background:**

Intestinal ligation is the cornerstone for damage control in abdominal emergency, yet it may lead to bowel ischemia. Although intestinal ligation avoids further peritoneal cavity pollution, it may lead to an increased pressure within the bowel segments and rapid bacterial translocation. In this study, we showed that severed intestine could be readily reconnected by using silicon tubes and be secured by using rubber bands in a canine model.

**Methods:**

Adult Beagle dogs, subject to multiple intestinal transections and hemorrhagic shock by exsanguination, randomly received conventional ligation vs. silicon tubes reconnecting (*n* = 5 per group). Intestinal transections were carried out under general anesthesia after 24-h fasting. The abdomen was opened with a midline incision. The small intestine was severed at 50, 100, and 150 cm below the Treitz ligament. Hemorrhagic shock was established by streaming blood from the left carotid artery until the mean arterial pressure reached 40 mmHg in 20 min. Fluid resuscitation and surgery began 30 min after the establishment of hemorrhagic shock. Severed intestines were ligated or connected with silicon tubes. Definitive repair was conducted in subjects surviving for at least 48 h.

**Results:**

Operation time was comparable between the two groups (39.6 ± 8.9 vs. 36.6 ± 7.8 min in ligation and reconnecting groups, respectively; *p* = 0.56). The time spent in managing each resection was also comparable (4.6 ± 1.1 vs. 3.8 ± 0.84 min; *p* = 0.24). Blood loss (341.2 ± 28.6 vs. 333.8 ± 34.6 ml; *p* = 0.48), and fluid resuscitation within the first 24 h (1676 ± 200.6 vs. 1594 ± 156.5 ml; *p* = 0.46) were similar. One subject in the ligation group was sacrificed at 36-h due to severe vomiting that led to aspiration. Four remaining dogs in the ligation group received definitive surgery, but two out of four had to be sacrificed at 24-h after definitive repair due to imminent death. All five dogs in the reconnecting group survived for at least a week. Radiographic examination confirmed the integrity of the GI tract in the reconnecting group. In both groups, plasma endotoxin concentration increased after damage control surgery, but the increase was much more pronounced in the ligation group. Microscopic examination of the involved segment of the intestine revealed much more severe pathology in the ligation group.

**Conclusion:**

The current study showed that the reconnecting resected intestine by using silicon tubes is feasible under emergency. Such a method could decrease short-term mortality and minimize endotoxin translocation.

## Background

In critically injured patients, primary repair of gastrointestinal (GI) tract injuries is often not feasible due to hemodynamic instability, coagulopathy, and metabolic acidosis [1–3]. In such cases, management of the GI tract injury is limited to the control of sepsis and hemorrhage [4–8]. Damaged intestines are excised, and the ends are simply ligated to prevent further contamination of the peritoneal cavity. Intestinal continuity is restored in subsequent definitive surgeries.

As the pressure inside the GI tract increases upon ligation, the intestinal wall becomes damaged, and the bacteria translocate into the systemic circulation [9]. Increased pressure also impedes blood supply to the intestine and aggravates the already existing damage [10]. In this study, we examined the effects of home-made silicon tubes to allow rapid damage control and to restore intestinal continuity in a Beagle dog model of multiple transection of small intestine and hemodynamic shock, with promising preliminary results.

## Methods

### Experimental design

Adult male Beagle dogs (*n* = 10.13–15 kg; Dasuo Biotech, Chengdu, China) were housed individually at 22–26 °C with 45–65% humidity. After 24-h fasting, dogs were anesthetized with ketamine (10 mg/kg; im). Induction was conducted with bolus i.v. pentobarbital injection (12–15 mg/kg) and was maintained at a speed of 1 mg/kg h. A central venous catheter was placed through the left external jugular vein for maintaining anesthesia and blood sampling. A catheter was placed in the right common carotid artery to monitor arterial pressure and heart rate.

**Fig. 1:**
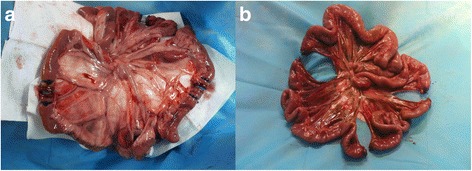
Representative small intestine segments after ligation (**a**) and reconnecting (**b**)

The abdomen was opened with a midline incision. The small intestine was severed at 50, 100, and 150 cm below the ligament of Treitz. Then the abdominal cavity was temporarily closed with towel forceps. Hemorrhagic shock was induced with bleeding from the jugular artery to maintain mean arterial pressure (MAP) at 40 mmHg in 20 min. Fluid resuscitation with Ringer’s solution was initiated 30 min after the hemorrhagic shock. Severed intestine was managed with conventional ligation or reconnection using a silicon tube (Xiangshu, Shanghai, China) (*n* = 5 for each, Fig. 1). The tube was inserted 2–3 cm into the edge of resection and was secured with rubber bands. A gastrostomy catheter (16 F) was placed for nutritional support later.

Definitive surgery was carried out at 48 h in dogs that survived beyond 48 h. Prior to definitive operation, the upper digestive tract was examined using imaging analysis of Meglumine Diatrizoate. 24 h after definitive surgery, the remaining animals in both groups were given nutritional support through a gastrostomy catheter (250 cal/day, in 250 ml volume).

### Outcome assessment

Plasma endotoxin concentration was determined by an enzyme-linked immunosorbent assay (ELISA) kits from Jinshanchuan (Beijing, China). Tissues were fixed in 10% formalin for 24 h and embedded in paraffin, and were processed for light microscopy with hematoxylin and eosin staining. For transmission electron microscopy (TME), tissues (at 50-cm distal to ligament of Treitz) were fixed in 4% glutaraldehyde and 1% paraformaldehyde, dehydrated, and embedded in Spurr resin. Ultrathin sections (2–3 mm) were stained with citrate. Photographs were obtained with a Philips EM208S transmission electron microscope (Philips; Eindhoven, Netherlands). The magnification of images was 10 × 1000.

### Statistical methods

Continuous variables are presented as means ± standard deviation and analyzed with Student’s *t* test or analysis of variance (ANOVA) of repeated measure with Statistical Package (SPSS 20.0 for windows, SPSS Inc., Illinois, USA). A probability of less than 0.05 was accepted as significant.

The Ethics Committee of Dongfang Hospital approved the study.

## Results

The current study showed practically zero short-term mortality in dogs receiving reconnecting after multiple transections of the small intestine, as well as much lower plasma endotoxin concentration.

Operation time of the damage control operation was comparable between the two groups (39.6 ± 8.9 vs. 36.6 ± 7.80 min in the ligation and reconnecting groups, respectively; *p* = 0.56). The time spent in managing each resection was comparable (4.6 ± 1.1 vs. 3.8 ± 0.8 min; *p* = 0.24). Blood loss (341.2 ± 28.6 vs. 333.8 ± 34.6 ml; *p* = 0.48) and fluid resuscitation within the first 24 h (1676 ± 200.6 vs. 1594 ± 156.5 ml; *p* = 0.46) were similar between the two groups.

**Fig. 2:**
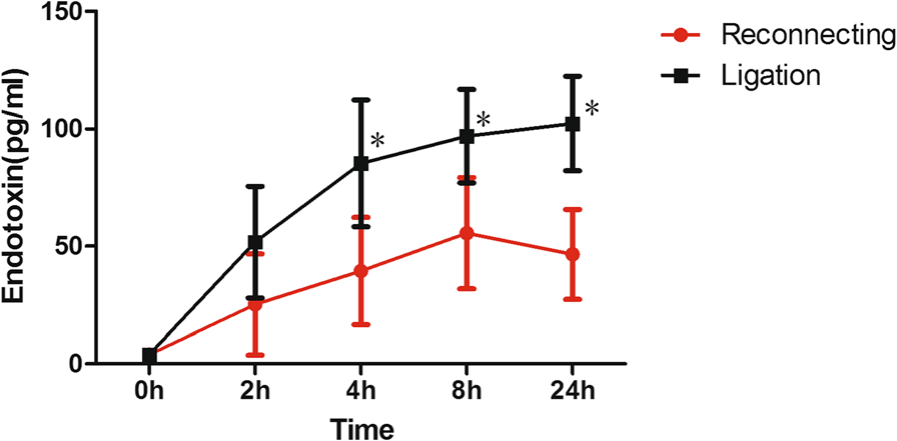
Plasma endotoxin concentration after ligation and reconnecting.^*^ refers to the *p* value between two groups was statistically significant

Plasma endotoxin concentration increased after the surgery in both groups, but was much more pronounced in the ligation group (Table 1; Fig. 2). One dog in the ligation group was sacrificed after 36 h due to severe vomiting that led to aspiration. The remaining four dogs in the ligation groups received definitive operation, but two had to be sacrificed within 24 h after definitive repair due to imminent death. Post-mortem analysis revealed large amount of ascites in abdominal cavity. All five dogs in the reconnecting group survived for at least a week after the definitive operation.

**Table 1:** Plasma endotoxin concentration after ligation and reconnecting

	0 h	2 h	4 h	8 h	24 h
Reconnecting	3.95 ± 0.75	25.19 ± 21.50	39.43 ± 22.86	55.55 ± 23.72	46.50 ± 19.22
Ligation	3.98 ± 0.60	51.78 ± 23.81	85.26 ± 26.89	96.89 ± 19.82	102.27 ± 20.03
*P* value	0.94	0.10	0.02*	0.01*	< 0.01*

Note: values were expressed as mean ± SD; ^*^ refers to the *p* value between two groups was statistically significant, ANOVA of repeated measures

**Fig. 3:**
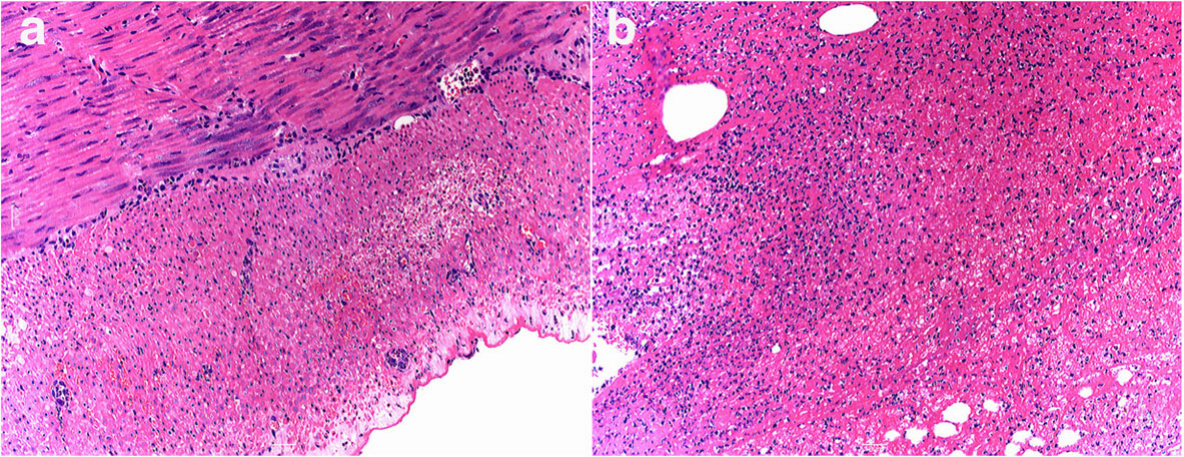
Intestinal mucosa (at 50-cm distal to ligament of Treitz) in dogs subjected to ligation (**a**) and reconnecting (**b**), at 48 h under light microspcopy (× 100)

**Fig. 4:**
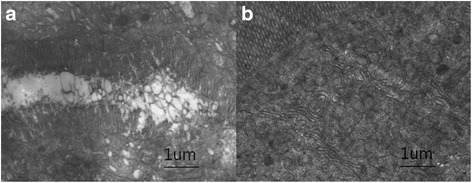
Transmission electron microscopy examination of the bowel (at 50-cm distal to ligament of Treitz) in dogs subjected to ligation (**a**) and reconnecting (**b**) at 48 h(10 × 1000)

**Fig. 5:**
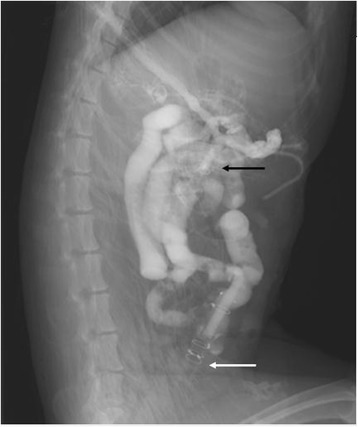
Radiographic examination of the upper digestive tract in a dog subjected to reconnecting at 48 h showed the contrast medium flowed smoothly through the intestine. There were no sign of air fluid level and swollen or obstructed intestines. Note: black arrow refers to proximal intestines; white arrow refers to distal intestines

Microscopic examination of the involved intestine revealed much more severe damage, including sloughing of surface epithelium, massive intraepithelial neutrophil infiltration and necrosis, in the ligation group vs. the reconnecting group (light microscopy in Fig. 3; TEM in Fig. 4). Radiographic examination of the upper digestive tract at 48 h in the reconnecting group showed the contrast medium flowed through the intestine (Fig. 5).

## Discussion

Patients with severe multiple enteric injuries often had severe comorbid conditions (such as hemodynamic shock). Currently, damage control surgery is to carry out ligation of severed/injuried GI tract [4, 7, 8]. Aggravated abdominal cavity contamination and bleeding can be easily controlled; however, continuing loss of fluids from the vascular compartment to the interstitial and third spaces leads to hemodynamic imbalance [11]. The ligations also elevate pressure within the bowel segments left in discontinuity and facilitate the development of pressure-related complications, such as bacterial translocation [12–14]. Consistent with a previous study [15], we observed rapid endotoxin translocation in the current study. Attempts have been made by previous studies to address the issue, such as an intraluminal drainage system to reduce intraluminal pressure [16]. Procedures required, however, are complex and time consuming.

In this study, we showed feasibility of reconnecting severed small intestine with silicon tubes. The method allowed rapid re-establishment of GI tract continuity. Comparison with ligation indicated that reconnecting could reduce bacteria translocation and tissue damage to small intestine in the involved segments. More importantly, short-term mortality (within a week after definitive surgery) is reduced to practically zero.

Time is of essence in definitive surgery; physicians must weigh risks vs. benefits [6, 17]. Ligation is considered a gold standard that could stabilize the physiological conditions of most patients. Definitive surgery, however, must be performed within a short period of time. Otherwise, patient’s condition will deteriorate [13, 18]. In the current series, we could complete the procedure within a very short period of time, without failure of this simple device (such as slipping-out of the tubes).

Another critical issue in the management of severe abdominal trauma is preserving the function of the gastrointestinal tract. Primary anastomosis could be hazardous because of the prolonged operation time and compromised hemodynamic conditions [4–7]. In this regard, temporally reconnecting transected intestines restored the continuity and the function of the GI tract, as evidenced by radiographic examination at 48 h after the reconnection.

We suspect in the future, this tubes system will be applied to patients with bowels ruptured, who be allowed for damage control surgical conditions. However, injuries in clinical settings are typically far more complex than those modeled in the current study. Clearly, the current study is a proof-of-concept in nature and requires further studies in animal studies before applying to human subjects.

## Conclusions

Reconnecting transected small intestine with silicon tubes and tuber bands is a viable method of damage control. Further studies are required to validate the results of this preliminary study.

## Abbreviations

GI: Gastrointestinal
MAP: Mean arterial pressure
TME: Transmission electron microscopy
